# Practical teaching in undergraduate human and dental medical training during the COVID-19 crisis. Report on the COVID-19-related transformation of peer-based teaching in the Skills Lab using an Inverted Classroom Model

**DOI:** 10.3205/zma001398

**Published:** 2021-01-28

**Authors:** Anne Röhle, Henrike Horneff, Marie-Christin Willemer

**Affiliations:** 1 Technische Universität Dresden, Faculty of Medicine Carl Gustav Carus, Medical Interprofessional Training Centre, Dresden, Germany; 2 University of Leipzig, Faculty of Medicine, Skills and Simulation Centre LernKlinik, Leipzig, Germany

**Keywords:** blended learning, e-learning, digitalisation, COVID-19, SARS-CoV-2, interactive learning, medical education, medical training, peer-assisted learning, skills lab

## Abstract

**Objective: **Drastic restrictions were imposed due to the COVID-19 pandemic, especially relating to the practical training part of the undergraduate human and dental medical training. During emergency mode teaching in the summer semester of 2020, a pilot project on practical classroom teaching under COVID-19 conditions was undertaken the Skills Lab Dresden, the Interprofessional Medical Training Centre (MITZ). Students were able to continue learning basic communication and manual skills. This project report presents the adaptations needed for this teaching concept and discusses their feasibility as well as selected evaluation results of the trial run.

**Description of the project:** In normal teaching, students rotate to complete training sessions in small groups. Teaching is provided in a peer-teaching format. An Inverted Classroom Model was implemented as a teaching concept during emergency operation with preparation through digital learning and classroom teaching. Organisational and teaching adjustments were carried out for the concept and to comply with containment regulations. The concept was evaluated by the students using a standardised online questionnaire.

**Results: **1012 students completed their training during emergency operation at the university. The containment regulations meant that there were a higher number of training sessions and a higher workload. Only one of the alternative dates provided had to be used for COVID-19-related reasons. Infection chains could be tracked. The majority of students found the communication of information via Moodle to be sufficient and did not experience any technical problems. An analysis of the students’ evaluation revealed a high level of overall satisfaction with the adapted teaching concept.

**Conclusion:** The MITZ will once again use the concept in a modified form should there be renewed or continued emergency operation. The Inverted Classroom Model will also be established as an integral part of regular teaching. The findings may be of interest to other Skills Labs to develop concepts for emergency operation teaching to efficiently utilise site-specific resources.

## Zusammenfassung

**Zielsetzung: **Bedingt durch die COVID-19-Pandemie entstanden insbesondere für die praktische Ausbildung des Human- und Zahnmedizinstudiums drastische Einschränkungen. Während der Notbetriebslehre im Sommersemester 2020 fand im Skills Lab Dresden, dem Medizinischen Interprofessionellen Trainingszentrum (MITZ), ein Pilotprojekt zur Umsetzung von praktischer Präsenzlehre unter COVID-19-Bedingungen statt. Studierende konnten weiterhin kommunikative und manuelle Basisfertigkeiten trainieren. Dieser Projektbericht stellt die nötigen Anpassungen für dieses Lehrkonzept vor und diskutiert deren Durchführbarkeit sowie ausgewählte Auswertungsergebnisse des Probedurchlaufs.

**Projektbeschreibung:** In der regulären curricularen Lehre absolvieren die Studierenden in Kleingruppen Trainingsstationen im Rotationsprinzip. Die Lehre findet im Peer-Teaching-Format statt. Für den Notbetrieb wurde als Lehrkonzept ein Inverted-Classroom-Modell mit Vorbereitung durch digitales Lernen und Präsenzlehre implementiert. Für das Konzept und die Einhaltung der Eindämmungsbestimmungen wurden organisatorische und didaktische Anpassungen vorgenommen. Mit Hilfe eines standardisierten Online-Fragebogens wurde die Studierendenevaluation durchgeführt.

**Ergebnisse:** Im Notbetrieb absolvierten 1012 Studierende die Trainings. Durch die Eindämmungsbestimmungen kam es zu einer höheren Anzahl an Trainingsterminen und einem erhöhten Arbeitsaufwand. Nur einer der bereitgestellten Ausweichtermine musste COVID-19-bedingt genutzt werden. Infektionsketten wären nachvollziehbar gewesen. Der Großteil der Studierenden empfand die Bereitstellung von Informationen über Moodle als ausreichend und sah keine technischen Probleme. Die Auswertung der Studierendenevaluation zeigte eine hohe Gesamtzufriedenheit mit dem angepassten Lehrkonzept.

**Schlussfolgerung: **Im erneuten oder fortzusetzenden Notbetrieb wird das MITZ das Konzept modifiziert wieder nutzen. Zudem wird das Inverted-Classroom-Modell als fester Bestandteil der regulären Lehre etabliert. Die Erkenntnisse können für andere Skills Labs zur Erarbeitung von Konzepten für die Notbetriebslehre von Interesse sein, um die standortindividuellen Ressourcen effizient zu nutzen.

## 1. Background

The Interprofessional Medical Training Centre (MITZ), the skills lab of the Faculty of Medicine Carl Gustav Carus Dresden, was faced with specific challenges with the TU Dresden’s decision to transfer from regular operation to emergency operation due to the COVID-19 pandemic. The curriculum of the undergraduate human medical training provides for 35 training sessions and the Dental Medicine degree 14 training sessions (see table 1 (Tab. 1)). Small groups of up to six students (ST) rotate between up to six sessions, each one lasting 50 minutes, on a single day of training. The sessions are supervised by peer tutors (PT) in a* peer-teaching format* [1]. Usually this means that 25-30 ST, five to ten PT, one to two simulated patients (SP) and the MITZ employees (MA) are on site. Following the announcement of the COVID-19 containment regulations, the employees reviewed the feasibility of different concepts, their potential learning success and compliance with the generally applicable hygiene measures and legal regulations [2], [3] to be able to offer practical teaching in the summer semester of 2020. A *corona concept* was developed as a pilot project using the* Inverted Classroom* (also known as the *Flipped Classroom* - ICM) teaching format. With the selected teaching method, the ST acquire knowledge in a digital preparation phase, which is then applied during the classroom session [4], [5]. Various e-learning models are used for this [6]. This report presents the adaptations necessary for teaching and discusses their feasibility as well as selected evaluation results from the trial run.

## 2. Description of the project

**Figure 1:**
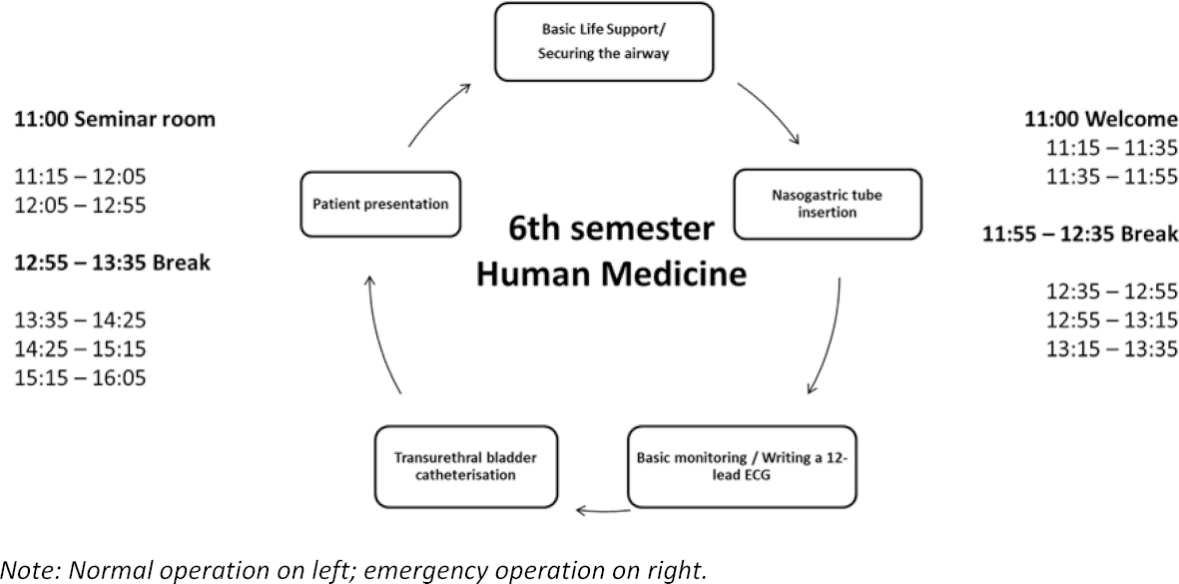
Abriged rotation principle using the example of a subject-related semester

A safety concept (see attachment 1 ) was developed based on the requirements of the State of Saxony [2], [3], the TU Dresden and the University Hospital Dresden crisis team. The reduced classroom times and digital teaching was intended to minimise the risk of infection and thus enable further training. The ICM learning scenario was chosen to utilise the existing resources of the MITZ, to make online teaching motivational, and use the time spent in the classroom on practical exercises. An abridged rotation principle was established (see figure 1 (Fig. 1)) based on regular teaching.

**Figure 2:**
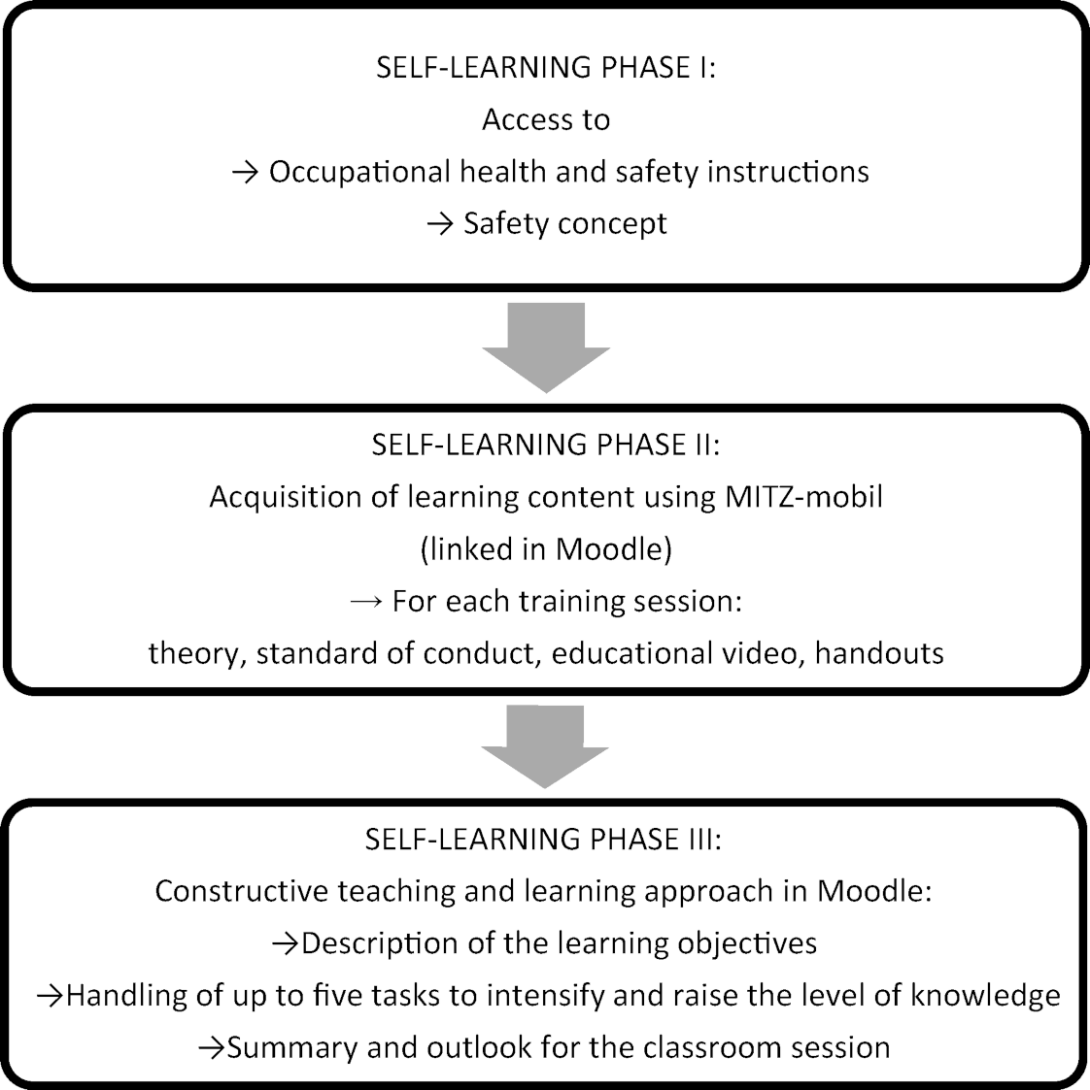
Structure of the preparatory e-learning content in the Moodle learning management system

At the MITZ, the ICM consists of classroom teaching and mandatory e-learning with three modules (see figure 2 (Fig. 2)). The ST prepare the learning content in the Moodle learning management system [7], [https://docs.moodle.org/39/de/Was_ist_Moodle] and apply it during the classroom session [8]. The MITZ-mobil online learning programme [9], [https://elearning.med.tu-dresden.de/mitz-mobil/], which has been available since 2015, has been integrated into Moodle. The learning content was accessed using various visualisation exercises and interactive exercises. ST received information about the ICM via the ePortal platform [10], which has been established since 2006.

Table 2 (Tab. 2) compares regular teaching with emergency teaching, illustrating the adjustments made.

The opinions of employees and ST were recorded to evaluate emergency mode teaching. A medical member of staff asked for positive and negative aspects, as well as aspects that could be improved, in ten 20-minute face-to-face interviews with employees. The ST evaluated the adapted teaching format with an online questionnaire based on EvaSys [https://www.electricpaper.de/]. Selected aspects from the questionnaire (see attachment 2 ) and the findings of the interviews are evaluated below.

## 3. Results

Emergency mode teaching in the MITZ took place from 4 May - 9 July 2020 for 1012 ST. Exploratory evaluation of the interviews with the employees revealed that there were no major problems. All the adjustments listed in table 2 (Tab. 2) were implemented. There was at least one alternative session available for each training session. During the first classroom teaching week, an alternative date had to be used due to one ST testing positive for COVID-19. On one occasion, no PT were invited, which is why the sessions had to be managed by employees at short notice. The necessary increase in the number of training courses has led to increased organisation and preparation of the ICM and hence an increased workload. In a few cases, the checkboxes in Moodle were incorrectly displayed to the employee during checking (see table 2 (Tab. 2)), although renewed login corrected the fault. The individual ST who had not prepared the e-learning content completed the teaching using tablet computers provided on site. Very few problems occurred with the implementation of the preparatory e-learning units. One group of ST had to return as they were given the wrong time. In some cases, not enough attention was paid to the time needed to get from live online events in the students’ home to classroom sessions in the MITZ. The survey assessment reveals a positive overall rating of the pilot project and the learning process by ST (see table 3 (Tab. 3)).

## 4. Discussion

The piloting of emergency mode teaching was successful. The organisation of the 2020 summer semester and the results of the evaluation show that the teaching adaptations were feasible and that the ST accepted the ICM very well. 

### Hygiene

The concept enabled classroom teaching and made it possible to trace and interrupt infection chains. However, the results do not permit any conclusions to be drawn about whether the concept would cope with rising infection rates.

#### Resources

The successful implementation of emergency mode teaching is based on the use of facility-specific resources (deployment of PT, rotation principle, extension of MITZ-mobil, structure expanded in Moodle, and training cycles) and their adaptation to new situations. MA were able to compensate for the additional workload through digital teaching, as many teaching projects were limited in emergency operation. 

#### Scheduling

Due to the extensive changes in the course of the entire faculty, PT schedules and ST timetables should be peer-examined several times in the current semester. Across the institution, travel times between classroom teaching and live online events, which are likely to be watched at home, increasingly need to be taken into account. 

#### Didactic concept

The ICM was successfully piloted. The majority of ST found the communication of information via Moodle to be sufficient and did not experience any technical problems. The ICM requires continuous support coupled with time-intensive supervision and further development by MA. When adapting the concept, care should be taken to differentiate between the digital teaching-based and classroom teaching-based learning objectives listed in Moodle, and also to integrate the new digital learning content into PT (post-) training [5]. The evaluation discussions only had an exploratory character. Qualitative research would be needed for more detailed information, for example by a systematic analysis of ST free comments or by conducting structured group interviews with the PT. Teaching should then be further developed together with the PT.

## 5. Conclusion

The MITZ will be able to once again use the Corona concept in a modified form should there be renewed or continued emergency operation. The ICM will also be established and further developed as an integral part of regular teaching. The findings may be of interest to other Skills Labs to develop concepts for emergency operation teaching to efficiently utilise site-specific resources. 

## Notes

Shared lead authorship by A. Röhle and H. Horneff. 

## Competing interests

The authors declare that they have no competing interests. 

## Supplementary Material

MITZ Safety Concept

EvaSys student evaluation questionnaire

## Figures and Tables

**Table 1:**
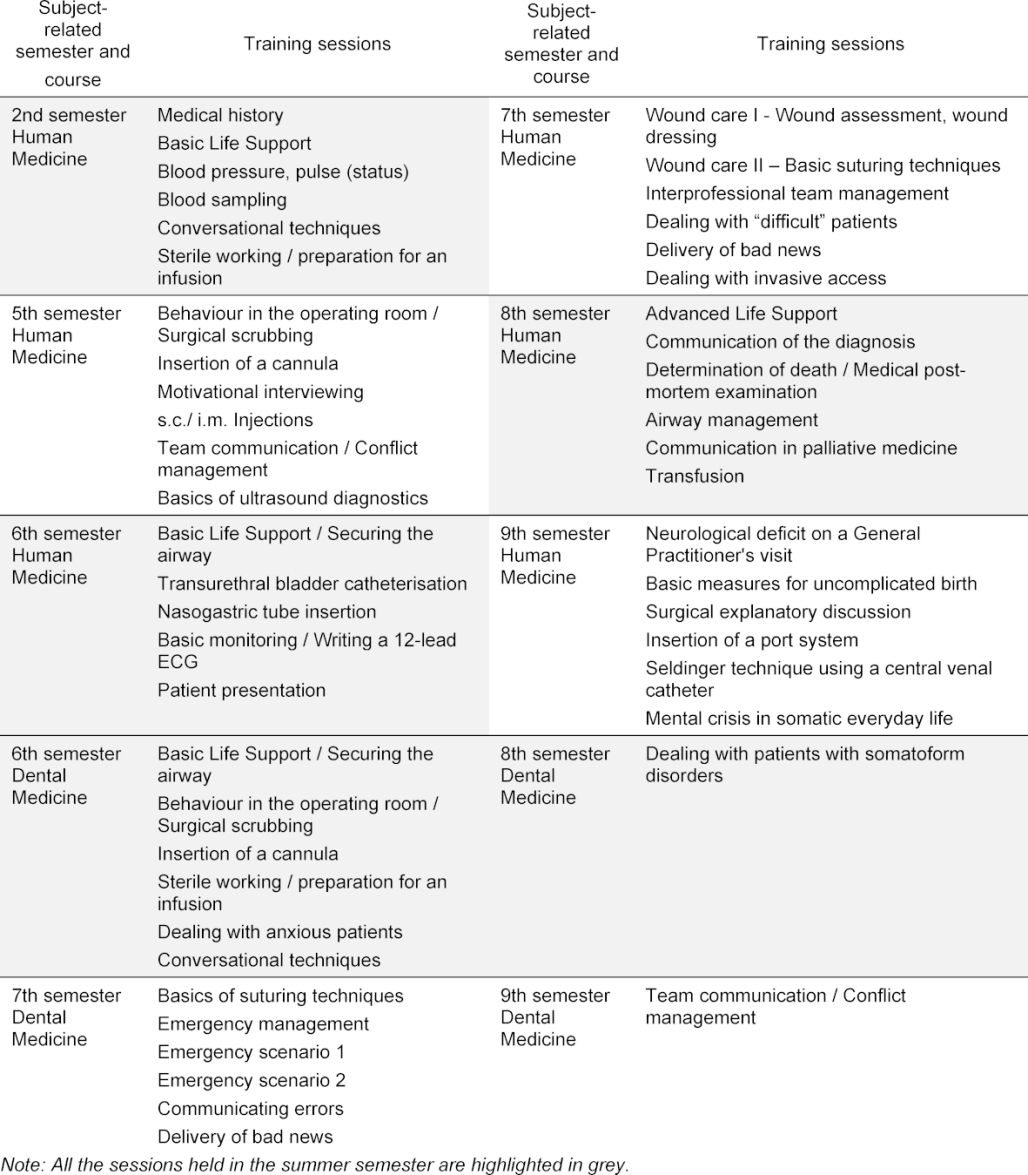
Overview of all training sessions in the MITZ

**Table 2:**
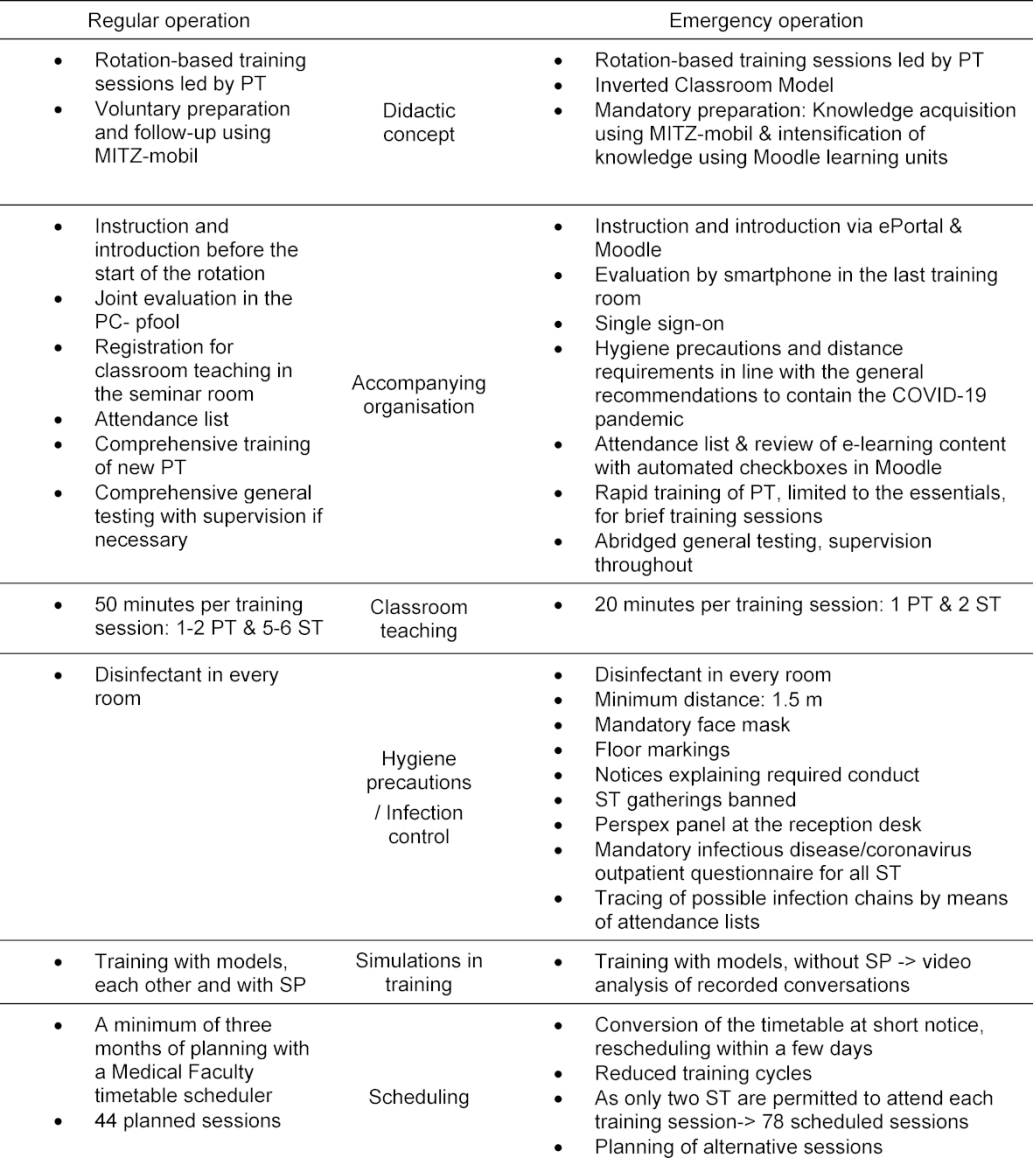
Comparison of regular teaching and emergency teaching in the MITZ

**Table 3:**
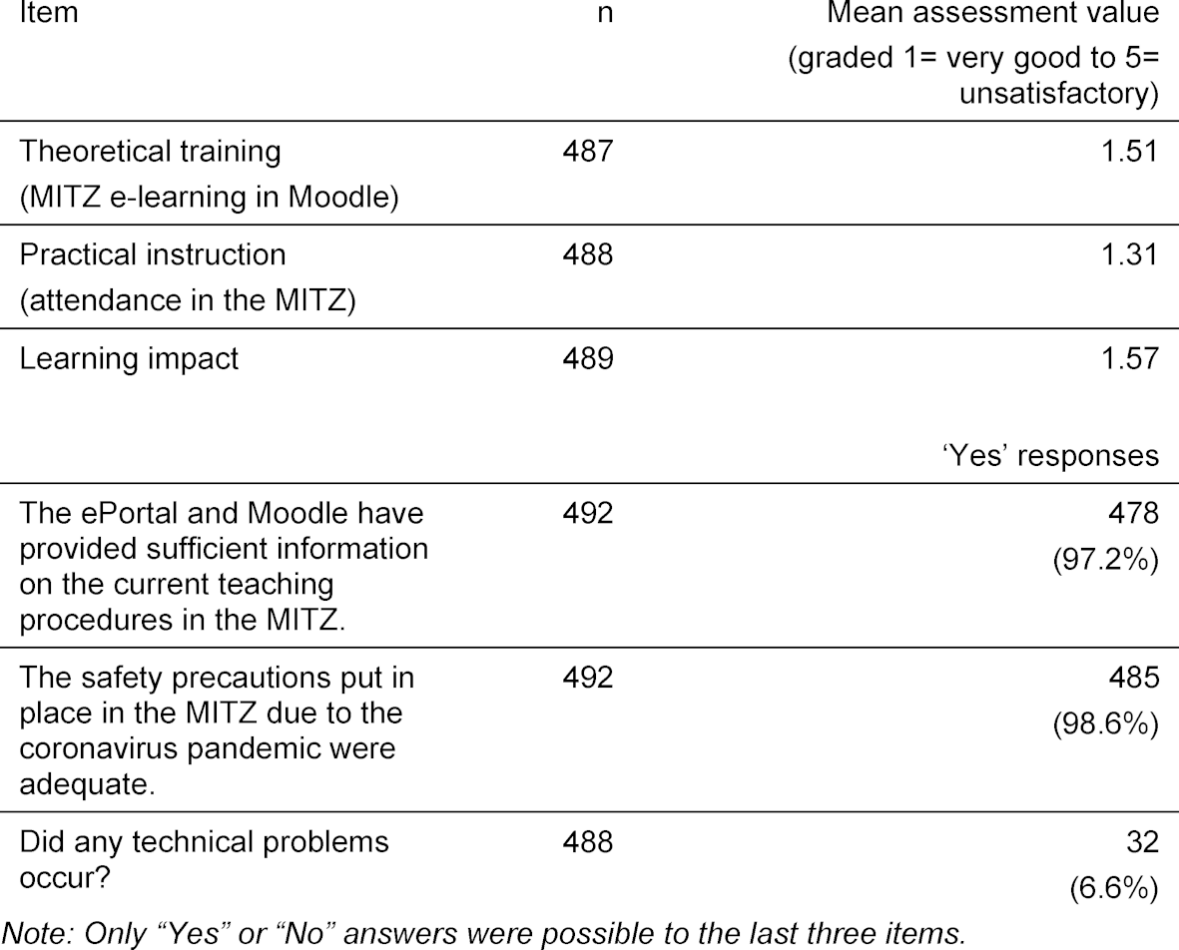
Evaluation of MITZ training in the summer semester 2020
