# Supplementary material for: Practical teaching in undergraduate human and dental medical training during the COVID-19 crisis. Report on the COVID-19-related transformation of peer-based teaching in the Skills Lab using an Inverted Classroom Model
Source: GMS J Med Educ. 2021 Jan 28;38(1):Doc2. doi: 10.3205/zma001398 (PMC7899122; doi:10.3205/zma001398)
Supplement: MITZ Safety Concept [file JME-38-1-2-s-001.pdf]

Attachment 1: *MITZ Safety Concept*

TECHNISCHE UNIVERSITÄT DRESDEN

Carl Gustav Carus Faculty of Medicine Teaching Department, Interprofessional Medical Training Centre

Safety concept for the organisation of classroom teaching in the 2020 summer semester for students of Human and Dental Medicine at the MITZ during the coronavirus pandemic

Dated: 14.05.2020

A safety concept for the MITZ has been developed due to the current development of the coronavirus pandemic. It has been developed in line with the Saxon Corona Protection Regulation dated 30 April 2020, the General Decree by the Saxon State Ministry of Social Affairs and Social Cohesion dated 17 April 2020 (Reference no. 15-5422/22) and the stipulations of the Vice-Rector for Academic and International Affairs for Education and International Affairs to restrict classroom teaching dated 20 April 2020.

The practical teaching content of MITZ training for students of Human and Dental Medicine can only be taught digitally to a limited extent (e-learning units via the Moodle learning platform). Therefore, in addition to digital teaching, abridged classroom teaching will be provided at the MITZ observing comprehensive safety precautions.

Depiction of classroom teaching under normal conditions:

The standardised training of practical basic medical skills under normal conditions is provided in small groups in rooms on the 1st and 2nd floors in Building 105. There are usually 3 to 5 students, one tutor and possibly a simulated patient in each room (Ø 15 m^2). The teaching day usually starts with instructions and information on fire and health protection in seminar room 248 (approx. 24 people within an area of 30 m^2) and ends with an evaluation in the PC-pool (18 m^2). A normal rotation training day at the MITZ consists of 5 to 6 different training sessions, each lasting 50 minutes.

Presentation of classroom teaching incorporating specific safety precautions to avoid the spread of coronavirus infection:

General measures:

- Students with cold symptoms or raised body temperature are prohibited from attending classes – should this be the case, it is imperative that they immediately consult a doctor or the coronavirus outpatient clinic
- Students should arrive just before the start of class and immediately leave the building at the end of the class
- Prior to the start of the class, the short medical history questionnaire will be collected, checked and sent off in line with the specifications
- Attendance lists will also be kept with the date and signature of the student
- A minimum social distance of 1.5 metres must be maintained between individuals
- The wearing of a face mask is mandatory for students and lecturers (preferably bring one with you, although the MITZ also has a stock of face masks)

Attachment 1 to: Röhle A, Horneff H, Willemer MC. *Practical teaching in undergraduate human and dental medical training during the COVID-19 crisis.* GMS J Med Educ. 2021;38(1):Doc2. DOI: 10.3205/zma001398

- Group sizes will be limited to 3 people per room (15 m^2, clinical semester) or 8 people per room (34-44 m^2, pre-clinical semester)
- A 15-minute break is allowed between classes to avoid an overlap between seminar groups arriving and departing
- Ensure hand hygiene (hand sanitiser dispenser in each classroom; the nearest washroom facility is signposted)
- There are also posters providing information on compliance with hygiene rules
- Floor markings have been put in place for class rotation to guide the flow of students and avoid crossings
- Separate WCs for employees and students
- No use of changing rooms: personal items must be kept with the individual at all times
- No patient contact in the MITZ
- There will be routine cleaning of surfaces and objects after each use (e.g. training dolls, medical equipment).
- Certified training/instruction of staff and student tutors regarding compliance/communication of hygiene rules and safety regulations (hand disinfection, frequent hand washing, avoidance of hand-face contact, room ventilation, regular breaks outside the building, no formation of small groups)
- A perspex panel has been fitted above the desk in the MITZ office 208 (MITZ Organisational Management)
- Flexible work scheduling and/or working from home for all MITZ employees to reduce the number of employees on site to a minimum
- The training session will be evaluated outside of the MITZ (at home or on a mobile device)

<u>Specific measures:</u>

- General information and instruction on the training day (hygiene regulations, safety precautions, fire and health protection, waste disposal) will be provided digitally through the Moodle learning platform and communicated through the e-portal
- Before the class, students will be explicitly advised that they should not attend classes at the MITZ if they have cold symptoms or a raised body temperature, and, if so, should consult a doctor or the coronavirus outpatient clinic
- The use of simulated patients (SP) is being critically examined and is expected only to occur in the 8th subject-related semester of Dentistry linked to a training session:
    - If the use of SP is indispensable for a classroom training session, they will not be used for physical examinations and only wearing a face mask and at a distance of 1.5 metres from the attendees.
- <u>Pre-clinical – Introduction to Clinical Medicine</u>
  2nd subject-related semester:
    - Medical history: video and video analysis
    - BLS: Training on doll – currently 3-4 students + 1 tutor per room (15 m^2)
    - Blood pressure/pulse: blood pressure on a fellow student, your own pulse
    - Blood sampling: on a model
    - Sterile work: no contact
    - Discussion techniques: video and video analysis
- <u>Clinical – Human Medicine</u>
  6th subject-related semester
    - BLS: training on doll
    - Transurethral bladder catheterisation: on a model

- Nasogastric tube insertion: on a model
- Basic monitoring/attachment of a 12-lead ECG: on a fellow student
- Patient presentation: case analysis and practical case discussion

8th subject-related semester

- ALS: training on doll
- Diagnostic communication: video and video analysis
- Medical post-mortem examination: training on doll
- Respiratory tract management: on a model
- Communication in palliative medicine: video and video analysis
- Transfusion: bedside test, preparation of an erythrocyte concentration (no contact)

▪ Clinical – Dental Medicine

6th subject-related semester

- BLS: training on doll
- Surgical scrubbing: no contact
- Insertion of a cannula: on a model
- Sterile work: no contact
- Dealing with anxious patients: video and video analysis
- Discussion techniques: video and video analysis

8th subject-related semester

- Treatment of somatoform disorders in dentistry: practical exercise and feedback session with simulated patient

▪ Optional classes

- Involve initiatives by students for students, with group sizes of up to 20 participants, which are divided into small groups with up to 5 participants
- For example, CaruSono, Wound management/Sutures, ALS, Trauma

- Currently cancelled → only available online
- They are to be made possible in compliance with the safety concept depending on the further development and in the urgent interest of preserving these groups, teaching new tutors etc.

Attachment 1 to: Röhle A, Horneff H, Willemer MC. *Practical teaching in undergraduate human and dental medical training during the COVID-19 crisis.* GMS J Med Educ. 2021;38(1):Doc2. DOI: 10.3205/zma001398
