# Supplementary material for: Practical teaching in undergraduate human and dental medical training during the COVID-19 crisis. Report on the COVID-19-related transformation of peer-based teaching in the Skills Lab using an Inverted Classroom Model
Source: GMS J Med Educ. 2021 Jan 28;38(1):Doc2. doi: 10.3205/zma001398 (PMC7899122; doi:10.3205/zma001398)
Supplement: EvaSys student evaluation questionnaire [file JME-38-1-2-s-002.pdf]

Attachment 2: *EvaSys Student Evaluation Questionnaire*

EvaSys	MITZ_Training_SoSe2020	Electric Paper
Department of teaching	MITZ	
MITZ	MITZ training	

Bitte so markieren: ☐ ☒ ☐ ☐ ☐ Please mark as follows

Korrektur: ☐ ■ ☐ ☒ ☐ Correction

General Questions

The ePortal and Moodle have provided sufficient information on the current teaching procedures in the MITZ. ☐ yes ☐ no

If not, to which issues would you have wanted (more) information on?

The safety precautions due to the coronavirus pandemic within the MITZ were adequate. ☐ yes ☐ no

If not, what was not adequate?

MITZ E-learning in Moodle

Did any technical problems occur? ☐ yes ☐ no

If yes, please explain which technical problems occured?

Global Evaluation of the MITZ Training in Grades

Theoretical training (MITZ e-learning in Moodle)	1 (very good)	☐	☐	☐	☐	☐	5 (unsatisfactory)
Practical teaching (classroom teaching in the MITZ)	1	☐	☐	☐	☐	☐	5
Learning impact	1	☐	☐	☐	☐	☐	5

Please use the section below to tell us what you liked most:

Please use the section below to share with us what you think still needs improving:

Attachment 2 to: Röhle A, Horneff H, Willemer MC. *Practical teaching in undergraduate human and dental medical training during the COVID-19 crisis.* GMS J Med Educ. 2021;38(1):Doc2. DOI: 10.3205/zma001398
